# Soft synthesis and characterization of goethite-based nanocomposites as promising cyclooctene oxidation catalysts†

**DOI:** 10.1039/d1ra04211d

**Published:** 2021-08-13

**Authors:** Andrei Cristian Kuncser, Ioana Dorina Vlaicu, Octavian Dumitru Pavel, Rodica Zavoianu, Mihaela Badea, Dana Radu, Daniela Cristina Culita, Arpad Mihai Rostas, Rodica Olar

**Affiliations:** National Institute of Materials Physics, Laboratory of Atomic Structures and Defects in Advanced Materials 405A Atomiştilor Str., Măgurele Ilfov 077125 Romania arpad.rostas@infim.ro; University of Bucharest, Faculty of Chemistry, Department of Organic Chemistry, Biochemistry and Catalysis 4-12 Regina Elisabeta Av. S3 Bucharest 030018 Romania; University of Bucharest, Faculty of Chemistry, Department of Inorganic Chemistry 90-92 Panduri Str. 050663 Bucharest Romania rodica.olar@chimie.unibuc.ro; Ilie Murgulescu Institute of Physical Chemistry 202 Splaiul Independentei 060021 Bucharest Romania

## Abstract

Goethite based nanocomposites with a different composition such as 6FeO(OH)·MnO(OH)·0.5H_2_O (Mn-composite), *x*FeO(OH)·M(OH)_2_·yH_2_O (Co-composite (M: Co, *x* = 12, *y* = 3), Ni-composite (M: Ni, *x* = 7, *y* = 2)) and *x*FeO(OH)·MO·yH_2_O (Cu-composite (M: Cu, *x* = 5.5, *y* = 3), Zn-composite (M: Zn, *x* = 6, *y* = 1.5)) have been prepared by a soft chemical synthesis consisting in acetate hydrolysis. The data provided by Fourier transform infrared (FTIR), ultraviolet-visible-near infrared (UV-Vis-NIR), electron paramagnetic resonance (EPR) and Mössbauer spectra account for a slight modification of all composites' physicochemical properties compared to the starting material. Powder X-ray diffraction and transmission electron microscopy (TEM) investigations revealed the secondary phase nature and presence along with that of goethite. The TEM data are also consistent with a nano rod-like morphology with a 5–10 nm width and an average length of 40 nm. The catalytic oxidation of cyclooctene with O_2_ using isobutyraldehyde as reductant and acetonitrile as a solvent was performed in batch conditions for 5 h at room temperature. The selectivity for the epoxide was higher than 99% for all tested solids. The conversion of cyclooctene decreased from 55% to 4% following the same order of variance as the base/acid sites ratio: Mn-composite > Fe-composite > Co-composite > Ni-composite > Zn-composite > Cu-composite. The 6FeO(OH)·MnO(OH)·0.5H_2_O (Mn-composite) exhibited the most promising catalytic activity in cyclooctene oxidation, which can be correlated with the redox ability of Mn(iii) combined with the increased base character of this solid. The catalytic activity of this sample decreases by 10% after several successive reaction cycles.

## Abstract 2

Goethite based nanocomposites with different compositions (6FeO(OH)·MnO(OH)·0.5H_2_O, *x*FeO(OH)·M(OH)_2_·*y*H_2_O or *x*FeO(OH)·MO·*y*H_2_O where M = Co, Ni, Cu or Zn) have been prepared by a soft chemical synthesis *via* acetate hydrolysis.

## Graphical abstract

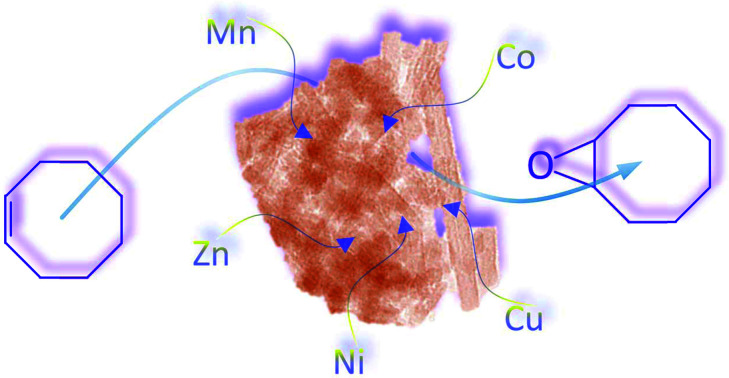

## Introduction

The low production costs and their chemical stability against UV radiation or corrosive agents make inorganic pigments one of the most important species of this type. Among these, both iron oxides and oxyhydroxides such γ-Fe_2_O_3_ (maghemite), Fe_3_O_4_ (magnetite), α-FeO(OH) (goethite), and FeO(OH)·H_2_O (limonite) are constituents of many paints, enamels, and varnishes.^1,2^ On the other hand, all ores enriched in such compounds represent the raw material for the cast or steel iron industry.^3,4^

Some concrete based on limonite or ilmenite–limonite is used as nuclear reactor shielding.^5–7^ Both goethite and limonite are used as precursors for preparing many functional materials such as magnetic oxides, maghemite, magnetite, and ferrites.^8–10^ Moreover, the limonite addition to lithium-ion batteries increases the ionic resistance and conductivity.^11,12^ A limonite-doped lithium borate glass was developed as gamma-ray shielding material.^13^

Natural limonite was also used as raw material to produce a nanosized zero-valent iron by hydrogen reduction with superior performance on *p*-nitrophenol decomposition compared with a commercial iron powder.^14^ Furthermore, a cotton fabric coated with a polymer containing a mixture of goethite, limonite, and hematite as additives exhibited bacteriostatic and antibacterial effects against *Staphylococcus aureus* and *Escherichia coli*.^15^

Far from these various applications, other studies use hydrated iron oxyhydroxide as a catalyst for inorganic and organic processes. Examples of such methods are arsenite oxidation,^16^ arsenic removal from wastewater,^17^ ammonia,^18^ pyridine^19^ or hydrogen sulphide^20^ removal from coke oven gas, coal liquefaction for oil production,^21^ organic compounds removing from wastewater^22–25^ and microcystin-LR hydrolysis in cancer prevention.^26^

On the other hand, iron-containing systems containing bimetallic^27–33^ or trimetallic layered oxyhydroxides^27,34–36^ were developed, based on their tunable electronic structures and rich active sites,^37^ as valuable nonprecious metal-based materials for oxygen evolution reaction (OER).

Synthesis of this kind of compounds with controlled size, shape, morphology, iron substitution, and the active surface is important. Moreover, since their properties are affected by all these factors, they also need to be precisely tuned depending on the requirements of particular applications.^38,39^

Usually, goethite is mainly obtained by the wet-chemical precipitation process starting from water-soluble iron salts (chloride, nitrate, or sulfate) by adding either caustic soda or ammonia in the presence of air as oxidizing agent.^40^ Parameters like pH, salt concentration, temperature, and stirring velocity are involved in the particle size and geometry, which control the properties.^38,39^

However, the attention in this field is now focused on the development of some non-conventional methods such as plasma treatment,^24,25^ electrodeposition,^27,30,31,34^ chemical deposition,^29,32^ or chemical deposition assisted by a magnetic field.^33^ Moreover, all these methods are eco-friendly and control both the nano-dimension of the particles and the substitution degree.

In the present work, we extended the study to obtain manganese, nickel, cobalt, copper, and zinc goethite-based nanocomposites by a soft chemical method successfully used for some ferrites synthesis.^41^ Furthermore, the influence of the second metallic ion from these nanocomposites on their catalytic behavior in cyclooctene epoxidation with molecular oxygen, in the presence of isobutyraldehyde, under ambient conditions was studied as well.

## Experimental part

### Reagents

We used high purity reagents purchased from Sigma-Aldrich (Saint-Louis, MO, USA) (FeCl_3_·6H_2_O, Mn(CH_3_COO)_2_·4H_2_O, Co(CH_3_COO)_2_·4H_2_O, Ni(CH_3_COO)_2_·4H_2_O, Cu(CH_3_COO)_2_·H_2_O, Zn(CH_3_COO)_2_·2H_2_O, ammonia 25%, H_2_O_2_) as received without further purification. Cyclooctene, isobutyraldehyde, and acetonitrile were also purchased from Sigma-Aldrich and have been previously distilled before reactions. Gaseous O_2_ with 99.99% purity purchased from Linde gas was used as an oxidant agent.

### Instruments and methods

Chemical analysis of iron, manganese, cobalt, nickel, copper, and zinc was performed using the usual micro methods^42^ after sample dissolution with hydrochloric acid. The iron was precipitated with ammonia and gravimetrically determined after calcination, while the other metallic ions were determined from the resulted solution. The heating curves (TG, DTG, and DTA) were recorded using a Labsys 1200 instrument (Setaram, Caluire, France), with a sample mass of about 20 mg over the temperature range of 293–1173 K, using a heating rate of 10 K min^−1^. The measurements were carried out in a synthetic air atmosphere (flow rate of 16.70 cm^3^ min^−1^) using alumina crucibles. The Fourier transform infrared (FTIR) spectra were recorded with a Spectrum BX II (Perkin Elmer, USA) spectrometer in the 350–4000 cm^−1^ range by accumulating 32 scans at a resolution of 4 cm^−1^. The powdered samples were diluted into KBr powder in a 1 : 100 mass ratio, ground thoroughly, and pressed into pellets. UV-Vis spectroscopy was performed in solid-state on a V 670 spectrophotometer (Jasco, Easton, MD, USA) with Spectralon as standard in the 200–1500 nm range. The electron paramagnetic resonance (EPR) spectroscopy measurements were carried out with a Bruker EMX premium X (Bruker, Karlsruhe, Germany) equipped with an X-SHQ 4119HS-W1 X-Band resonator at a microwave frequency of 9.4457 GHz and power of 0.06325 mW. Further measurements parameters were: conversion time 10 ms, time constant 5.12 ms, modulation amplitude 0.3 mT with one scan. We used a digital temperature control system ER 4131VT with a liquid nitrogen cryostat from Bruker (Bruker, Karlsruhe, Germany) for cooling. The ^57^Fe Mössbauer spectra have been obtained in transmission geometry, at 6 K and room temperature, by inserting the samples in a close cycle Janis cryostat (Edina, Minnesota, USA). A SEECO-type spectrometer (Edina, Minnesota, USA) operating under the constant acceleration mode and a ^57^Co(Rh) radioactive source of about 30 mCi activity were used. The acquired ^57^Fe Mössbauer spectra were analyzed using the NORMOS software, which allows the decomposition of the measured absorption pattern in spectral components corresponding to different iron non-equivalent positions. In the case of a continuous distribution of the hyperfine parameters, the fitting procedure can be performed accordingly using specific routines that provide the envisaged probability distribution function and complementary average hyperfine parameters. The isomer shift is reported relative to the isomer shift of metallic Fe at room temperature. Powder X-ray diffraction (XRD) patterns were recorded with a Bruker D8 Advance X-ray diffractometer (Bruker, Karlsruhe, Germany) (Cu anode and Ni filter, *λ* = 1.54184 Å) in Bragg–Brentano configuration. We determined the lattice parameters and the average crystallites size by the Rietveld refinement method^43^ using the MAUD software. The JEOL 2100 Transmission Electron Microscope (TEM) (Tokyo, Japan), equipped with energy dispersive X-ray (EDS), has been used for transmission electron microscopy investigations. Specimens have been prepared using the standard powder method.

### General procedure for goethite and goethite-based nanocomposites synthesis

The goethite was synthesized by adding 10 mL solution 25% NH_3_ to a solution of 10 g FeCl_3_·6H_2_O in 100 mL distilled water. The reaction mixture was stirred at room temperature for 1 h until a brown precipitate formed. The solid product was filtered off, washed several times with water, and air-dried.

The syntheses of goethite-nanocomposites were performed by adding 20 mmol freshly prepared goethite to solutions containing 10 mmol M(CH_3_COO)_2_·*n*H_2_O (M: Mn, Co, Ni, Cu, and Zn) in 100 mL water. Reaction mixtures were heated at 373 K under continuous stirring for 24 h. The obtained brown products were filtered off, washed several times with water, and air-dried.

### Textural characterization

Nitrogen adsorption–desorption isotherms at 77 K were recorded on a Micromeritics ASAP 2020 automated gas adsorption system (Norcross, GA, USA). The samples were degassed at 90 °C for 12 hours under vacuum before analysis. Specific surface areas (*S*_BET_) were calculated according to the Brunauer–Emmett–Teller (BET) equation, using adsorption data in the relative pressure range between 0.05 and 0.30. The total pore volume (*V*_total_) was estimated from the amount adsorbed at the relative pressure of 0.99. The pore size distribution curves were obtained from the adsorption data using a DFT (density functional theory) model.

### Catalytic tests

This study evaluates the catalytic activities of the synthesized materials in the oxidation of cyclooctene with molecular oxygen. We used isobutyraldehyde as a reductant and acetonitrile as a solvent in batch conditions. Thus, 20 mg of material were contacted with 0.04 mol cyclooctene and 0.08 mol isobutyraldehyde in 10 mL acetonitrile as a solvent. We performed the catalytic tests for 5 h at room temperature. Molecular oxygen was admitted in a sealed stirred flask of 250 mL by a tube linked to an oxygen pressurized cylinder employing a manometer. During the tests, the oxygen pressure was maintained at 1 atm. Before reactants and the catalyst admission in the reactor, the air was removed by purging molecular oxygen for 5 minutes. During the reaction, at each hour, quantities of 50 μL were extracted from the reactor and analyzed with a Gas Thermo Quest Chromatograph (ThermoFisher Scientific Inc., Waltham, MA, USA) equipped with an FID detector and a capillary column with DB5 stationary phase. All products, as well as reactants, were identified by comparison with standard samples (retention time in GC) and by mass spectrometer-coupled chromatography VARIAN SATURN 2100 T (LabX, Midland, ON, Canada) to evaluate the cyclooctene conversion and the selectivity to epoxide.

### Base sites determination

The total number of base sites was determined using the irreversible adsorption of acrylic acid (p*K*_a_ = 4.2). Samples of dried solids (0.05 g) were contacted for 2 hours (duration required for reaching the equilibrium in the liquid–solid system) with 10 mL of 0.01 M solution of acrylic acid in cyclohexane in brown sealed bottles under mild stirring (150 rpm) at room temperature. It was assumed that the interaction of the solids with atmospheric CO_2_ and water was negligible since the samples were exposed to the atmosphere only during weighing. The concentration of the acrylic acid remaining in the solution after reaching equilibrium was determined by UV-Vis spectrometry at *λ*_max_ = 225 nm using the Jasco V-650 spectrometer (Tokyo, Japan). For each solid sample, 3 parallel determinations were performed, and the obtained results were averaged. The amount of acrylic acid (AA) adsorbed was calculated with the formula:AA_i_ − AA_f_ = AA_ads_where indexes i and f refer to the initial amount and the final amount of acrylic acid in the solution, respectively.

The method is inspired by the one used to determine base sites in hydrotalcite-type materials.^44^ However, in the case of these solids, we could not use phenol for the separate determination of strong base sites since phenol is known to give colored combinations with iron.

The total concentration of base sites was calculated with the formula:*C*_B_ = AA_ads_/wt [mmoles AA/gram of sample]where wt is the weight of the solid sample.

### Acid sites determination

The total number of acid sites was determined by pyridine adsorption. The distribution of acid sites, Lewis and Brönsted, respectively, were calculated from the areas of the corresponding peaks in the DRIFT spectra recorded on FT/IR-4700 Jasco spectrometer (Tokyo, Japan). Samples of dried solids (0.05 grams) were contacted with pyridine aliquots (0.2 μL each) and maintained under inert flow at 90 °C for the removal of physisorbed pyridine. The procedure was repeated until the weight of the sample after two consecutive additions of pyridine was constant (did not vary with more than 0.0001 g). Then, the DRIFT spectrum of the sample with adsorbed pyridine was recorded, considering the DRIFT spectrum of the freshly dried solid as background. According to literature data,^45–47^ the bands corresponding to pyridine adsorbed on Lewis acid sites appear in the ranges of 1435–1455 and 1570–1615 cm^−1^ while those corresponding to pyridine adsorbed on Brönsted acid sites appear in the range of 1520–1555 and at 1630 cm^−1^. In the ESI,† we have included Fig. S1† with the DRIFT spectra of the pyridine adsorbed on the samples.

## Results and discussions

In this paper, we report the synthesis and characterization of some goethite based nanocomposites of type: 6FeO(OH)·MnO(OH)·0.5H_2_O (Mn-composite), *x*FeO(OH)·M(OH)_2_·*y*H_2_O (Co-composite (M: Co, *x* = 12, *y* = 3) and Ni-composite (M: Ni, *x* = 7, *y* = 2)) and *x*FeO(OH)·MO·*y*H_2_O (Cu-composite (M: Cu, *x* = 5.5, *y* = 3) and Zn-composite (M: Zn, *x* = 6, *y* = 1.5)), respectively. These nanocomposite materials were synthesized by the transition ion acetates hydrolyze in an aqueous suspension of freshly prepared FeO(OH)·H_2_O as presented in reactions (1)–(3). This process is accompanied by Mn(ii) to Mn(iii) oxidation and M(OH)_2_ into MO transformation in the case of Cu(ii) and Zn(ii). These samples were characterized as nanocomposites by chemical and thermal analysis, IR, UV-Vis-NIR, EPR, and Mössbauer spectroscopy. Simultaneously, their morphology and particle dimension was provided by powder X-ray diffraction and TEM studies. Textural parameters were calculated from N_2_ sorption isotherms.

### Chemical analysis and thermal decomposition

We deduced the composition of these species from the metal contents, the mass of water lost up to 423 K (*W*_1_), the mass of water lost up to 633 K (*W*_2_), and residue formed at 873 K during the thermal decomposition, as reported in Table 1.

**Table 1:** Analytical and thermal data for goethite-based nanocomposites

Compound (notation)	% Fe		% M		% residue		% *W*_1_		% *W*_2_	
	Calc.	Exp.	Calc.	Exp.	Calc.	Exp.	Calc.	Exp.	Calc.	Exp.
6FeO(OH)·MnO(OH)·0.5H_2_O (Mn-composite)	53.18	53.07	8.96	8.53	88.56	89.03	1.43	1.10	10.01	9,87
12FeO(OH)·Co(OH)_2_·3H_2_O (Co-composite)	55.24	55.19	4.86	4.75	85.16	85.31	4.45	4.42	10.39	10.27
7FeO(OH)·Ni(OH)_2_·2H_2_O (Ni-composite)	52.07	52.00	7.82	7.68	84,39	84.58	4.80	4.71	10.80	10.71
5.5FeO(OH)·CuO·3H_2_O (Cu composite)	49.36	49.22	10.21	10.15	83.36	83.51	8.68	8.60	7.96	7.89
6FeO(OH)·ZnO·1.5H_2_O (Zn composite)	52.29	52.14	10.20	10.02	87.37	87.54	4.21	4.15	8.42	8.31

The thermal decomposition of all samples occurs in two endothermic steps. The first step corresponds to crystallization water elimination, which occurs up to 413–523 K, followed immediately by the same compound elimination due to oxyhydroxide/hydroxide decomposition.

### IR spectroscopy investigations

Iron oxyhydroxides, FeO(OH)·*n*H_2_O consists of a vast array of oxo and hydroxo groups with a three-dimensional structure in which the layers are connected through hydrogen bonds. The basic units in these polynuclear species that generate specific bands in the IR spectra of goethite consist of groups of two or three Fe(iii) ions links by oxo or hydroxo bridged units. Essential bands noticed in the spectra of synthesized goethite-based nanocomposites are summarized in Table 2. The infrared spectra are presented in Fig. S2 in the ESI.†

**Table 2:** Absorption maxima in IR spectra of the samples (the notations: vs – very strong, s – strong, m – medium, and w – weak describes the signal intensities)

FeO(OH)	Mn-composite	Co-composite	Ni-composite	Cu-composite	Zn-composite	Assignment
3410 m	3410 m	3410 m	3420 m	3380 m	3410 m	*ν*_asym_(H_2_O)
3140 s	3130 s	3130 s	3130 s	3140 m	3140 m	*ν*_sym_(H_2_O)
1640 w	1640 w	1640 w	1600 w	1645 w	1620 w	*δ*(H_2_O)
1140 w	1115 w	1125 w	1130 vw	1120 w	1115 w	*δ*(M-OH)
890 s	895 m	895 s	895 s	890 m	900 m	*ρ*_r_(OH_2_)
800 s	800 m	800 s	795 s	800 m	795 m	
610 s	605 m	600 s	605 s	600 m	605 m	*ρ*_w_(OH_2_)
455 vs	460 vs	460 vs	450 s	460 vs	460 vs	*ν*(M–O)
410 vs	410 vs	410 vs	410 vs	410 vs	410 vs	

In the 3130–3410 cm^−1^ region, one can notice two broad bands assigned to *ν*(OH) asymmetric and symmetric stretching vibration modes for lattice water. The bands around 1600 (*ν*(OH)), 890, 800 (*ρ*_r_(OH_2_)), and 600 cm^−1^ (*ρ*_w_(OH_2_)) are also due to the water molecule vibration modes. Moreover, the last ones indicate that some of these molecules are coordinated to the metallic centers. The hydroxo groups are distinguished from the aqua ones by the appearance of bands in the range 1115–1140 cm^−1^ assigned to *δ*(M–OH). Two bands assigned to metal–oxygen stretching vibrations appear in all spectra in the 400–460 cm^−1^ range.^48^

1 6FeO(OH)·H_2_O + Mn(CH_3_COO)_2_ + 0.5O_2_ →6FeO(OH)·MnO(OH)·0.5H_2_O + 2CH_3_COOH + 3.5H_2_O

2 *x*FeO(OH)·H_2_O + M(CH_3_COO)_2_ → *x*FeO(OH)·M(OH)_2_·*y*H_2_O + 2CH_3_COOH + (*x* − *y* − 2)H_2_O (M: Co, Ni)

3 *x*FeO(OH)·H_2_O + M(CH_3_COO)_2_ → *x*FeO(OH)·MO·*y*H_2_O + 2CH_3_COOH + (*x* − *y* − 1)H_2_O (M: Cu, Zn)

### UV-Vis-NIR spectroscopy characterization

The UV-Vis-NIR spectroscopy proved to be a valuable tool to establish the oxidation state of transition ions. We used the goethite itself for baseline calibration and as a reference to eliminate the iron interference. In this condition, this method revealed only the d–d bands characteristic for the second transition metallic ion present in the composite network. Thus, the Mn-composite exhibits two bands at 9130 and 14 925 cm^−1^. These are assigned to spin allowed transitions ^5^B_1g_ → ^5^B_2g_ and ^5^B_1g_ → ^5^A_1g_ in an octahedral stereochemistry associated with [Mn(iii)O_6_] chromophore. The bands at 9660 and 17 860 cm^−1^ for Co-composite arise from ^4^T_1g_ → ^4^T_2g_ and ^4^T_1g_ → ^4^T_1g_ spin allowed transitions in an octahedral stereochemistry of [Co(ii)O_6_] chromophore. The similar chromophore [Ni(ii)O_6_] is responsible for the bands at 10 310 and 23 256 cm^−1^ assigned to ^3^A_2g_ → ^3^T_2g_ and ^3^A_2g_ → ^3^T_1g_ transitions, respectively. The large and unsymmetrical band located at 10 200 cm^−1^ for Cu-composite accounts for a square-planar [Cu(ii)O_4_] chromophore and, as a result, can be assigned to d_*xy*_ → d_*x*^2^−*y*^2^_ transition.^49^

### EPR spectroscopy characterization

**Fig. 1:**
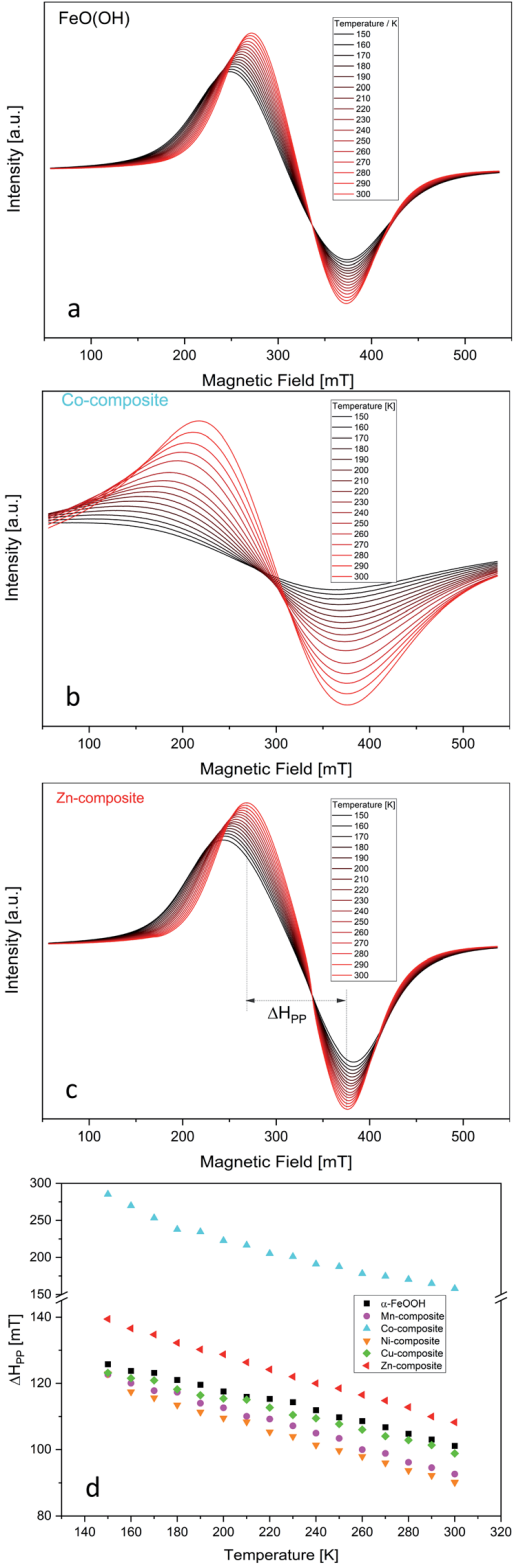
Temperature dependency of the EPR spectra for FeO(OH) (a), Co-composite (b) and Zn-composite (c). Temperature dependency of the peak-to-peak line width of the EPR spectra of the goethite and goethite-based nanocomposites (d). The temperature was varied in 10 K per step in the range 150–300 K.

We recorded each compound's EPR spectra at different temperatures beginning with 150 K being increased in 10 K steps until 300 K. The obtained characteristic spectra are presented in Fig. 1(a–c) for goethite, Co-composite, and Zn-composite samples. The EPR spectra for the other goethite-based nanocomposites are shown in the ESI (Fig. S3†).

The very broad EPR signal observed for all samples highlights the FeO(OH) strong ferromagnetic properties at low temperature. By increasing the temperature, the ferromagnetic properties vanish slowly, which is pointed out in Fig. 1d. The peak-to-peak line width of the EPR signal is plotted against the temperature. After reaching the Curie temperature, the material would become paramagnetic, and the peak-to-peak line width would present no changes. Since the Curie temperature of α-FeO(OH) is located at about 900 K,^50^ this point was not reached in this study.

Regarding the changes in the peak-to-peak linewidths of the EPR spectra as a function of the temperature, it is clear that the used elements influenced the ferromagnetic behavior of the FeO(OH). Fig. 1d summarizes these changes, where cobalt induces the most significant changes in ferromagnetic behavior. The other elements presented in the composite network cause small but visible changes. Thus, the peak-to-peak line width temperature dependency is similar to the FeO(OH)·H_2_O but different. This indicates a shift in the Currie temperature of the composites.

### Mössbauer spectroscopy characterization

**Fig. 2:**
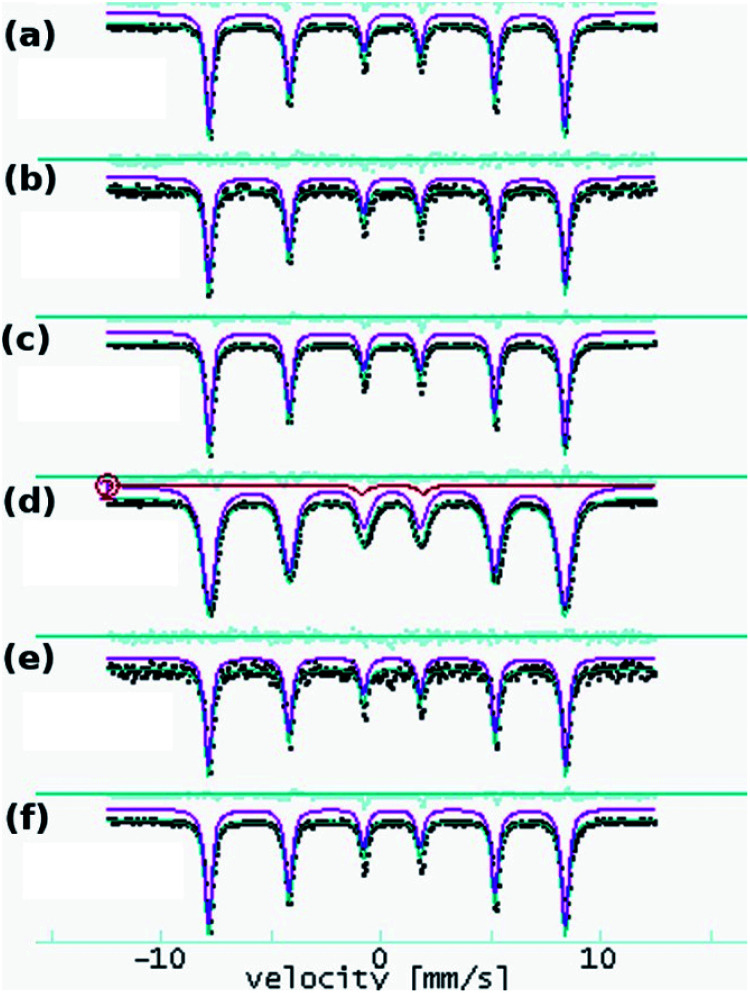
^57^Fe Mössbauer absorption spectra at 6 K (black dots) and fit results (purple line) obtained on goethite and goethite nanocomposite samples: FeO(OH) (a), Cu-composite (b), Zn-composite (c), Mn-composite (d), Ni-composite (e) and Co-composite (f).

Due to the preparation conditions, a substitution effect of goethite can be considered. Therefore, we recorded the Mössbauer spectra of both goethite and goethite-based nanocomposites have to confirm or exclude this effect. Mössbauer absorption spectra obtained at 6 K on FeO(OH) and nanocomposite samples are shown in Fig. 2.

All the spectra show a sextet pattern characteristic to α-FeO(OH) at low temperatures as the main component. The spectra have been processed at room temperature with distributions of ^57^Fe positions and low temperature with distinct ^57^Fe positions.

**Fig. 3:**
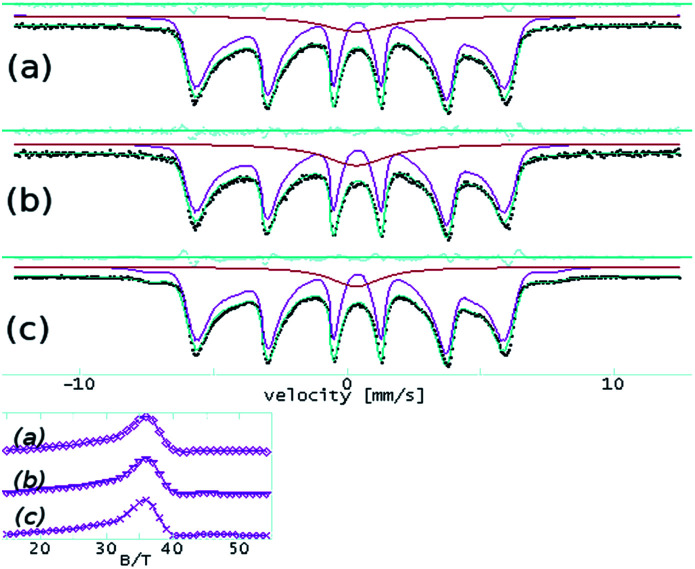
^57^Fe Mössbauer spectra at room temperature on FeO(OH) (a), Mn-composite (b) and Co-composite (c). The inset shows corresponding hyperfine field distributions, with a maximum at ∼36 T, consistent with the α-FeO(OH) hyperfine field at room temperature.

The room temperature experiments provide only ESI,† and the results are shown in Fig. 3.

The effects related to the presence of Mn(iii), Co(ii), Ni(ii), Cu(ii), and Zn(ii) in the α-FeO(OH) structure have been followed according to the variation of the hyperfine parameters. The fit results are shown in Table 3. The quadrupole splitting (QUA) and the magnetic hyperfine field BHF values are in good agreement with specific values of α-FeO(OH). The isomer shift (ISO), as well as the spectral line width (WID), do not show significant variations among the investigated samples.

**Table 3:** Fit parameters on the investigated samples

Sample/parameters	FeO(OH)	Cu-composite	Zn-composite	Mn-composite	Ni-composite	Co-composite
ISO	0.37	0.37	0.37	0.37	0.37	0.37
QUA	−0.24	−0.24	−0.23	−0.24	−0.24	−0.24
BHF	50.3	50.3	50.3	50.2	50.4	50.4
WID	0.35	0.35	0.36	0.35	0.38	0.38
ISO		0.52				
QUA		2.79				
WID		0.53				

In the case of the Mn-composite, the additional presence of a quadrupole doublet can be associated with a small fraction of very fine FeO(OH) nanoparticles in a magnetic relaxation regime. According to this specific evolution of the Mossbauer hyperfine parameters (almost unchanged among the analyzed samples), we conclude that the structure of the α-FeO(OH) remains unaffected by the presence of the second transition metallic ion.

### X-ray powder diffraction

**Fig. 4:**
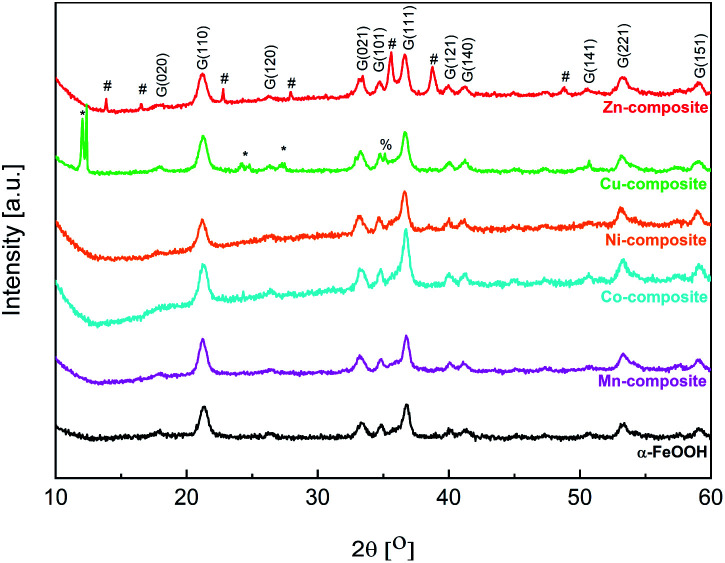
Powder X-ray diffractograms for goethite and goethite-based nanocomposites (G – goethite, * – copper(ii) acetate, # – ZnO (wurtzite), % – CuO (tenorite)).

The main crystalline phase was identified for all samples as α-FeO(OH) (PDF. No. 1008766). Fig. 4 shows the X-ray powder diffractograms for goethite and goethite-based nanocomposites.

From the Rietveld refinement data (Table 4), it can be observed that the mean crystallite size varies slightly between the goethite and the goethite-based nanocomposites. Such an effect may appear if a doping process of the goethite is considered. Moreover, except for Zn-composite, a slight increase in the lattice parameters can be observed for the other goethite-based nanocomposites. The Zn-composite lattice parameters are approximately the same as for FeO(OH)·H_2_O. As shown in Fig. 4 and Table 4, for the Cu-composite and Zn-composite, along with the α-FeO(OH) phase, another phase(s) appears. In the X-ray diffractogram of Cu-composite, a CuO phase (tenorite) was identified based on the JCPDS no. 1526990, with the lattice parameters determined by Rietveld refinement analysis as presented in Table 4. Based on the two intense diffraction peaks at small angles (12°) and the other three peaks at 24.2, 24.8, and 27.3°, copper(ii) acetate (JCPDS no. 00-027-1126) can be identified along with goethite and copper(ii) oxide. The X-ray diffractogram of Zn-composite contains diffraction peaks assigned to the ZnO phase with a hexagonal structure based on the JCPDS no. 89-1397.

**Table 4:** Rietveld refinement data for goethite and goethite-nanocomposites

Sample	Lattice parameters *a*, *b*, *c* (Å)	Mean crystallite size (nm)
FeO(OH)	4.598 ± 0.001	17.9 ± 0.31
	9.945 ± 0.002	
	3.017 ± 0.0006	
Mn-composite	4.609 ± 0.0008	19.40 ± 0.43
	9.976 ± 0.002	
	3.011 ± 0.0005	
Co-composite	4.604 ± 0.001	19.40 ± 0.43
	9.950 ± 0.002	
	3.019 ± 0.0006	
Ni-composite	4.620 ± 0.001	20.97 ± 0.43
	9.977 ± 0.002	
	3.024 ± 0.0007	
Cu-composite FeO(OH) 88%	4.617 ± 0.0009	20 ± 0.34
	9.955 ± 0.002	
	3.024 ± 0.0005	
CuO 12%	4.684 ± 0.001	89.36 ± 1.878
	3.421 ± 0.0006	
	5.139 ± 0.001	
Zn-composite	4.588 ± 0.001	19.58 ± 0.18
	9.941 ± 0.003	
	3.016 ± 0.0007	

### Transmission electron microscopy investigations

**Fig. 5:**
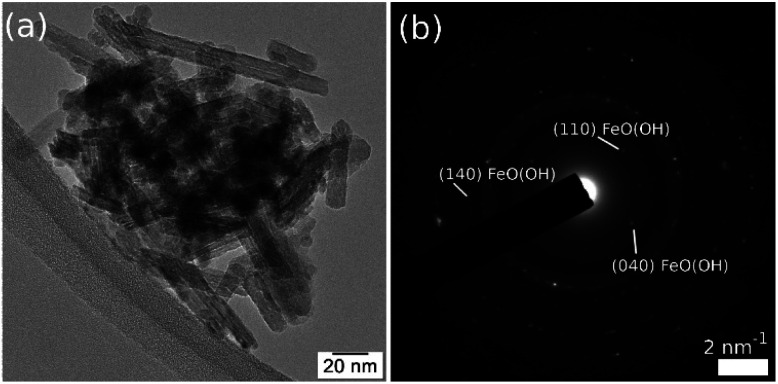
TEM image (a) and corresponding SAED (b) on FeO(OH) sample.

In Fig. 5, the TEM image and corresponding diffraction pattern acquired on FeO(OH) sample, as a reference, show a nano rod-like morphology and the α-phase of FeO(OH) (CIF 1008766). The crystalline nanorods have a relatively uniform width (5–10 nm) and a large distribution of lengths, with an average of roughly 40 nm.

**Fig. 6:**
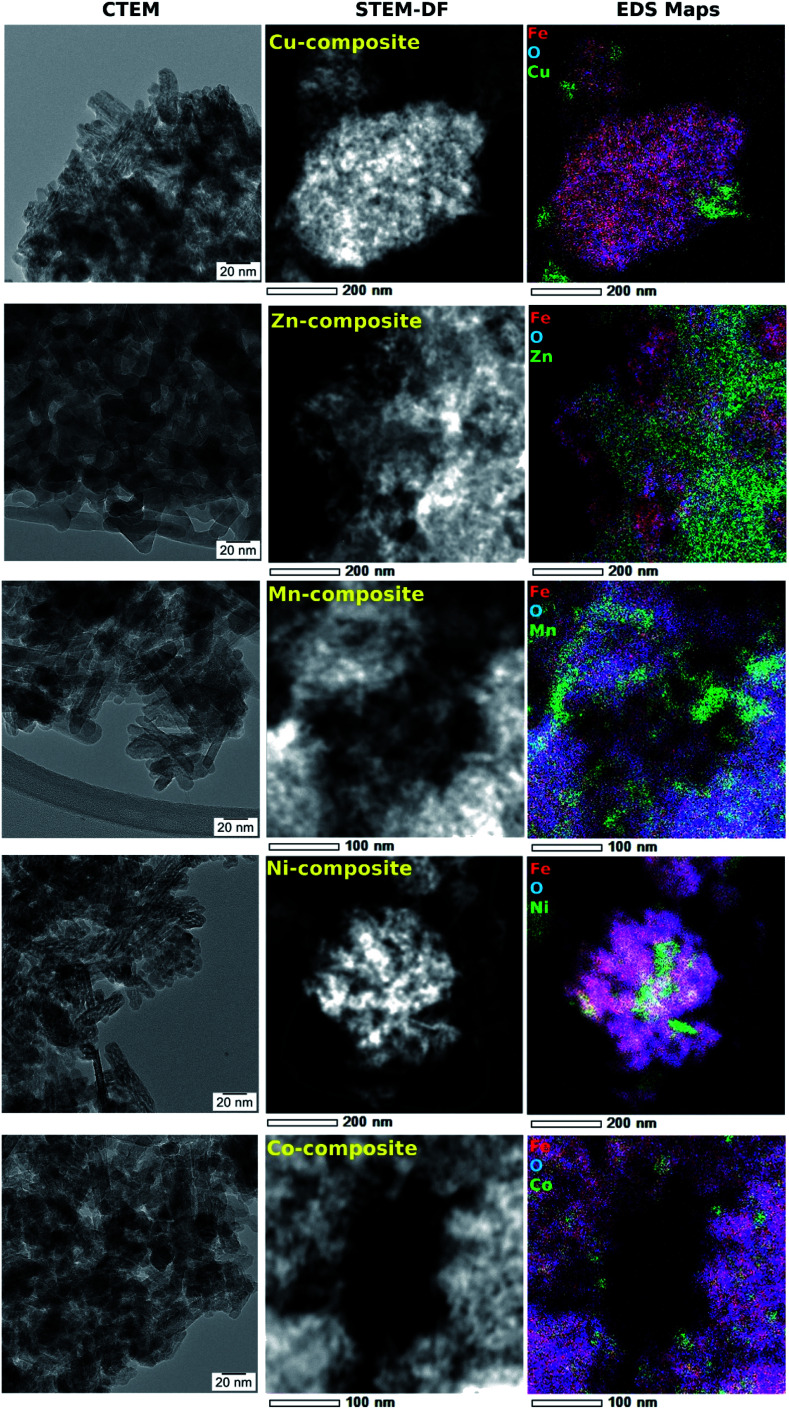
A comparative TEM study of goethite-based samples.

**Fig. 7:**
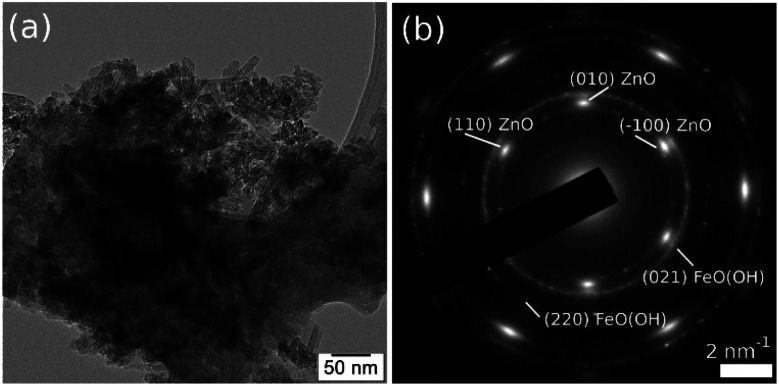
TEM image (a) and SAED pattern (b) obtained on Zn-composite sample, showing the formation of a highly textured ZnO secondary phase, oriented in (001) zone axis.

The FeO(OH) phase's morphology tends to be preserved along with the entire series of samples, as shown in Fig. 6. It is worth mentioning that the ∼20 nm size as provided by Rietveld analysis for the FeO(OH) phase in all cases is a very rough estimation, given the elongated morphology of the entities. The energy-dispersive X-ray spectroscopy (EDX) maps in Fig. 6 shows a specific aggregation of the doping element for all the nanomaterials. In the case of Cu-composite, the TEM investigations correlated with Rietveld refinement suggest the formation of a CuO (JCPDS no. 526990, tenorite) secondary phase. In the case of the Zn-composite, the elemental mapping (Fig. 6), the TEM image, and electron diffraction (Fig. 7, a and b) show the presence of a relatively thin layer of highly textured ZnO along with the familiar FeO(OH) phase. This solves the issue regarding the unidentified secondary phase from the Rietveld refinement mentioned above.

For the rest of the samples (Mn-composite, Ni-composite, and Co-composite), the elemental mappings analyzed in correlation with the powder X-ray diffraction results suggest the formation of incoherent Mn(iii), Ni(ii), respectively Co(ii) rich entities.

### Textural characterization

**Fig. 8:**
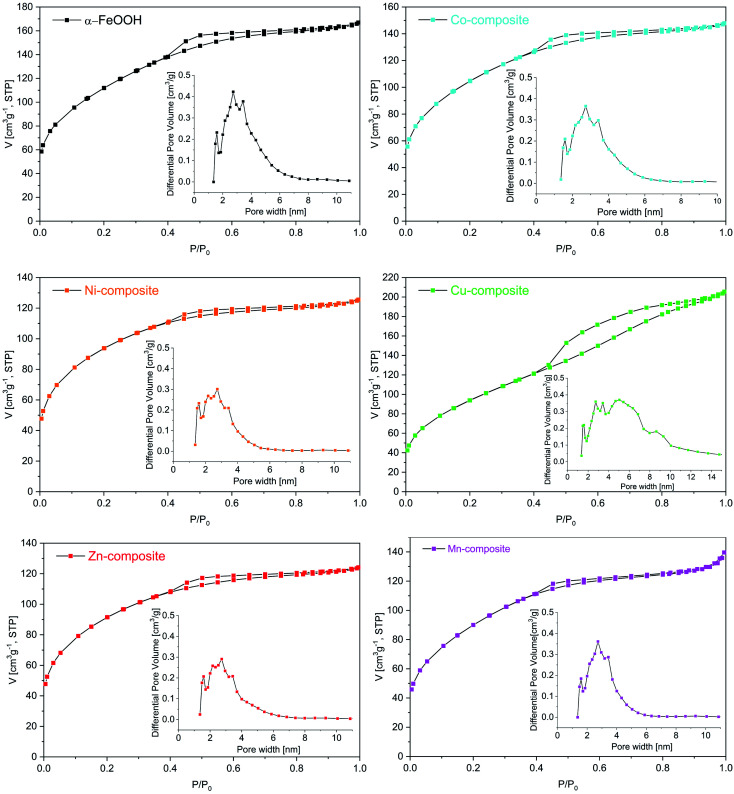
N_2_ adsorption–desorption isotherms of the samples (inset: pore size distribution).

Textural characterization of the samples was carried out by N_2_ adsorption–desorption analysis. According to the IUPAC classification, all the isotherms (Fig. 8) are of type IV.^51^ It can be noticed that in the region of low relative pressures (*p*/*p*_0_) up to 0.4, the amount of N_2_ adsorbed increases sharply. As *p*/*p*_0_ rises above 0.4, the uptake of N_2_ is slower, while at *p*/*p*_0_ values higher than 0.8, the adsorption curve tends to flatten. All isotherms display H2 hysteresis loops due to capillary condensation in mesopores, whose area differs depending on the pore size. The pore size distribution (insets of Fig. 8) obtained using a DFT model is multimodal. The average pore size is around 3 nm for goethite and M-composites (M: Mn, Co, Ni and Zn), while for the Cu-composite, it is 4.05 nm. The goethite has a specific surface area (*S*_BET_) of 394 m^2^ g^−1^, while for the composites, the *S*_BET_ is lower but over 300 m^2^ g^−1^ (Table 5). The obtained *S*_BET_ values are much higher than those reported by other authors for similar materials.^52^

**Table 5:** Textural parameters of the samples

Sample	*S*_BET_ (m^2^ g^−1^)	Pore volume (cm^3^ g^−1^)	Average pore size (nm)
α-FeO(OH)	394	0.258	3.03
Co-composite	364	0.228	2.95
Ni-composite	321	0.194	2.91
Cu-composite	342	0.317	4.05
Zn-composite	314	0.192	2.95
Mn-composite	320	0.215	3.20

### Catalytic activity

In addition to the large numbers of chemical reactions that show a peculiar scientific and economic interest, oxidation plays a crucial role. Selective oxidation, in particular selective oxidation of alkenes, is by far the most widely used method by which oxygen atoms can be inserted into molecules without leading to mineralization.^53^ Epoxides are important functional intermediates playing a crucial role in pharmaceuticals, pesticides, cosmetics, and materials production.^54^ However, the selective oxidation of alkenes requires complexes based on homogeneous^55–57^ or heterogeneous^58–61^ catalysts. Almost all of these catalysts have been used in the presence of non-environmentally friendly oxidizing agents such as NaClO,^62^ PhIO,^63^ dioxiranes,^64^*tert*-butylhydroperoxide,^65^ or potassium peroxomonosulfate.^66^ Hydrogen peroxide or molecular oxygen are viable alternatives to those described above. Up to date, there are several studies concerning epoxidation of alkenes in aerobic conditions using transitional metal complexes/support as catalysts and isobutyraldehyde as reductant, which are carried out in mild conditions.^67,68^

Total conversion of cyclooctene to the corresponding epoxide was recently reported for catalysts consisting of Co(ii) complexes with Schiff base ligands supported on silica-coated magnetite tested under reflux conditions.^69^ However, the synthesis of such catalysts is difficult and expensive, and the energy consumption for heating the reaction mixture is high. Another expensive catalyst that allowed reaching 85% conversion of cyclooctene with 92% selectivity for epoxide at a lower temperature (*e.g.*, 35 °C) is a Co(ii) coordinated metal–organic framework.^70^ Co_3_O_4_ nanoparticles encapsulated in the inside wall of a *meso*-SiO_2_ shell, which allowed reaching 90% conversion at 40 °C,^71^ manganese doped cerium oxide catalysts, which allowed getting 80% conversion at 100 °C,^72^ were recently reported as highly active heterogeneous catalysts for cyclooctene epoxidation using isobutyraldehyde and molecular oxygen. The disadvantages of the latter two systems are that the synthesis of Co_3_O_4_ encapsulated in *meso*-SiO_2_ shell catalyst is difficult to control.

**Scheme 1:**
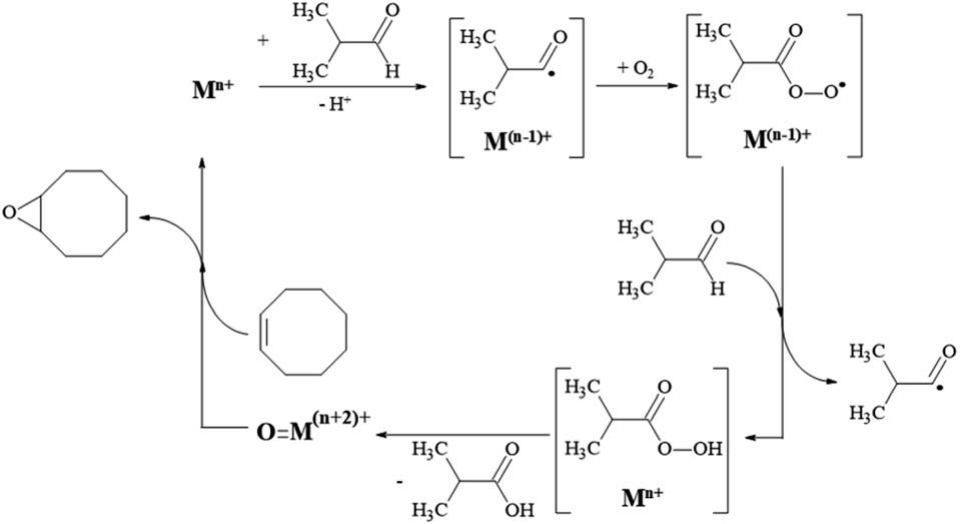
The reaction mechanism for cyclooctene oxidation with O_2_ in the presence of isobutyraldehyde.

In contrast, in the case of Mn-doped cerium oxide catalysts, the reaction conditions involve high energy consumption. Until now, iron oxide hydroxide (FeOOH) was used as an efficient catalyst only for alcohol oxidation, organic sulfide oxidation, and epoxidation of alkenes in the presence of H_2_O_2_^73^ while the catalytic activity of FeOOH based nanocomposites containing MnO(OH), Co(OH)_2_, Ni(OH)_2_, CuO or ZnO in the epoxidation of cyclooctene with molecular oxygen has not been reported. Such catalytic systems could present interest since their synthesis is not difficult and does not imply high costs. The reaction mechanism of cyclooctene oxidation with molecular oxygen in the presence of isobutyraldehyde is analogous to that suggested by Nam and co-workers.^74^ It involves several steps which confirm that the generation of isobutyric acid and the epoxide formation is in a linear dependence (Scheme 1). However, for all the investigated samples, the epoxide selectivity is more than 99%, confirming the unique character of this selective oxidation.

**Fig. 9:**
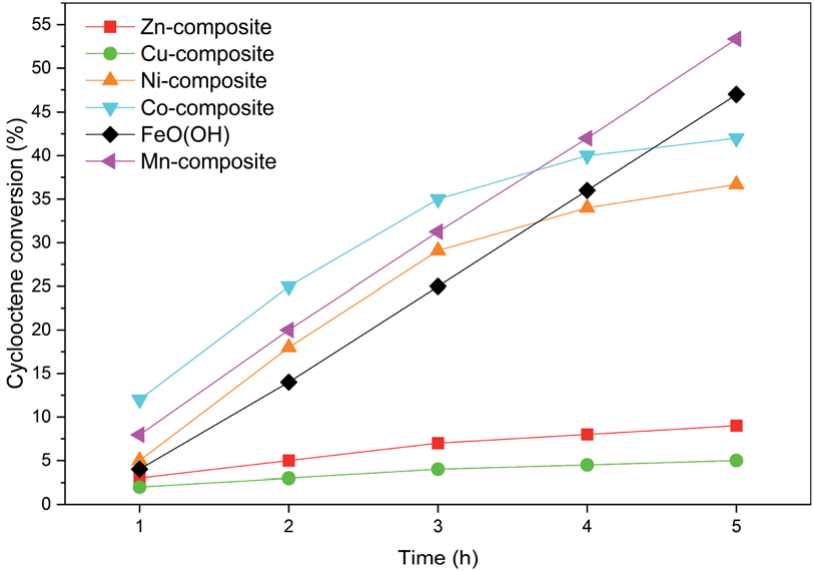
The samples activities after 5 h in a cyclooctene oxidation reaction.

However, several factors limit or improve catalytic activities due to the presence of three various trends. They depend on the cations in the composition of the solids. The lowest cyclooctene conversions of up to 10%, where a plateau level was quickly reached, were obtained with the Zn-composite and Cu-composite samples (Fig. 9). Both Zn(ii) and Cu(ii) cations do not display a large domain of oxidation states, allowing the cations' oxidation–reduction abilities. This characteristic is in agreement with Nam's conclusions,^74^ claiming that the oxidation of olefins occurs in the presence of high-valent oxometal intermediates produced by the reaction of the peroxyacid with the metallic ions from the catalyst. Ni-composite and Co-composite show a similar variation trend while the plateau level of the conversion is reached after 4 hours. However, the Co-containing catalyst displays better activities than the Ni-containing one due to its better ability to play the oxidation-reduction role.

The goethite (FeO(OH)·H_2_O) and Mn-composite show higher activity values than the other samples. The linear dependence of conversion values *versus* the reaction time is noticeable. Even after 5 hours, the appearance of a conversion level plateau is not reached.

Considering the result of the acid and base sites determinations presented in Table 6, there is a clear correlation between the ratio of base sites/acid sites and the conversion values obtained with different catalysts after 5 hours. The manganese presence leads to the best conversion values, which can also be related to the fact that it induces an increased base character of the solid (base sites/acid sites ratio = 4.44).

**Table 6:** Acid–base properties of the investigated samples

Composite	Mn	Fe	Co	Ni	Cu	Zn
Total acidity (mmoles Py per g)	0.9	1.6	1.5	1.7	1.6	1.2
% Bronsted	21	18.1	37.9	0.0	6.7	14.5
% Lewis	79	81.9	62.1	100.0	93.3	85.5
Total basicity (mmoles AA per g)	3.9	5.3	3.7	3.5	0.5	0.7
Ratio base sites/acid sites	4.44	3.33	2.40	2.11	0.31	0.59

Moreover, according to the mechanism presented in Scheme 1, the first step in cyclooctene transformation consists of metal ion reduction. Taking into account the ionization potential (*E*) associated with each cation presented in the composites (*E*(Mn^2+^/Mn^3+^), 33.69 eV; *E*(Co^+^/Co^2+^), 17.03 eV; *E*(Ni^+^/Ni^2+^), 18.03 eV; *E*(Cu^+^/Cu^2+^), 20.29 eV and *E*(Zn^+^/Zn^2+^), 17.89 eV),^75^ it is evident that the reverse transformation will involve the same energy but with an opposite sign. This means that the Mn-composite will provide for the first oxidation step higher energy than the other ones.

Hence, the synergistic effect of the ability to perform oxidation-reduction cycles and the increased base character induced by the involved modifying cations leads to better materials for selective oxidation of olefins. The small fraction of very fine FeOOH nanoparticles detected by Mössbauer spectra in the Mn-composite sample could also account for its improved activity. The results obtained with the Mn-composite at room temperature were better than those reported for cyclooctene epoxidation using other simple catalytic systems such as Ni/nanoporous carbon (*e.g.*, 50% conversion).^68^

**Fig. 10:**
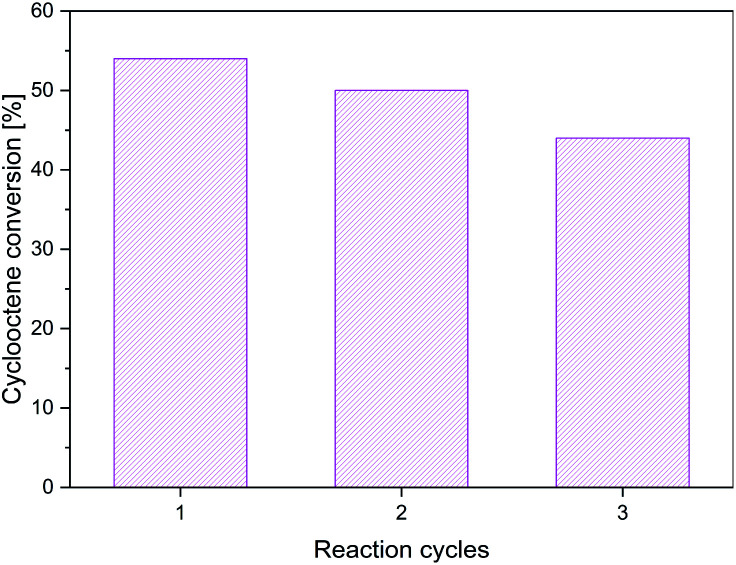
The cyclooctene conversion in the presence of Mn-composite after three reaction cycles.

The recycling studies for the best material, *i.e.*, MnFe_2_O_*x*_, indicated its reasonable stability since, after three reaction cycles, the conversion decreases only by 10% (Fig. 10). The decrease of activity from cycle to cycle could be due to the adsorption of the isobutyric acid generated as a by-product on the base sites of the catalyst since the catalyst was only dried and not washed with solvent before being reused.

## Conclusions

New goethite-based composites containing Mn(iii), Co(ii), Ni(ii), Cu(ii), and Zn(ii) were prepared and characterized from a physicochemical and morphological point of view. The Mössbauer spectra evidenced only the goethite pattern even at low temperatures. In contrast, the different ferromagnetic behavior evidenced by EPR spectra and the characteristic d–d bands is consistent with the second transition ion presence in the composite network. The lattice parameters vary only slightly from a composite to other. Powder X-ray diffraction and TEM data indicate a CuO and ZnO phase in the corresponding composite. On the other hand, the elemental mappings suggest the rich entities based on Mn(iii), Ni(ii), and Co(ii) for the different composites. These data are also consistent with the nano rod-like shape of these materials. The catalytic activity for the selective oxidation of olefins is due to the effect of the redox ability of the modifying cations in the goethite-based nanocomposites and base sites/acid sites ratio. The stability of the modified goethites is sensible due to the action of the isobutyric acid generated as a by-product. The composites will be studied in other catalytic reactions considering that such materials that act at room temperature are easy to synthetized and does not imply high costs. To improve the catalytic abilities, these will be calcined, and the new oxide-based composites will also be tested from this point of view.

## Author contributions

Andrei Cristian Kuncser: conceptualization, data curation, formal analysis, investigation, validation, writing – original draft, writing – review & editing, Ioana Dorina Vlaicu: onceptualization, data curation, formal analysis, investigation, validation, writing – original draft, writing – review & editing, Octavian Dumitru Pavel: data curation, formal analysis, investigation, writing – original draft, Rodica Zavoianu: conceptualization, data curation, formal analysis, investigation, supervision, writing – review & editing, Mihaela Badea: conceptualization, data curation, formal analysis, investigation, Dana Radu: data curation, formal analysis, investigation, validation, writing – original draft, Daniela Cristina Culita: data curation, formal analysis, investigation, Arpad Mihai Rostas: conceptualization, data curation, formal analysis, investigation, validation, writing – original draft, writing – review & editing, Rodica Olar: conceptualization, data curation, formal analysis, investigation, supervision, validation, writing – original draft, writing – review & editing.

## Conflicts of interest

There are no conflicts to declare.

## Supplementary Material

RA-011-D1RA04211D-s001
