# Supplementary material for: Soft synthesis and characterization of goethite-based nanocomposites as promising cyclooctene oxidation catalysts
Source: RSC Adv. 2021 Aug 13;11(44):27589–602. doi: 10.1039/d1ra04211d (PMC9037824; doi:10.1039/d1ra04211d)
Supplement: RA-011-D1RA04211D-s001 [file RA-011-D1RA04211D-s001.pdf]

Electronic Supplementary Material (ESI) for RSC Advances.
This journal is © The Royal Society of Chemistry 2021

Soft synthesis and characterization of goethite-based nanocomposites as promising cyclooctene oxidation catalysts

Andrei Cristian Kuncser, Ioana Dorina Vlaicu, Octavian Dumitru Pavel, Rodica Zavoianu, Mihaela Badea, Dana Radu, Daniela Cristina Culita, Arpad Mihai Rostas and Rodica Olar

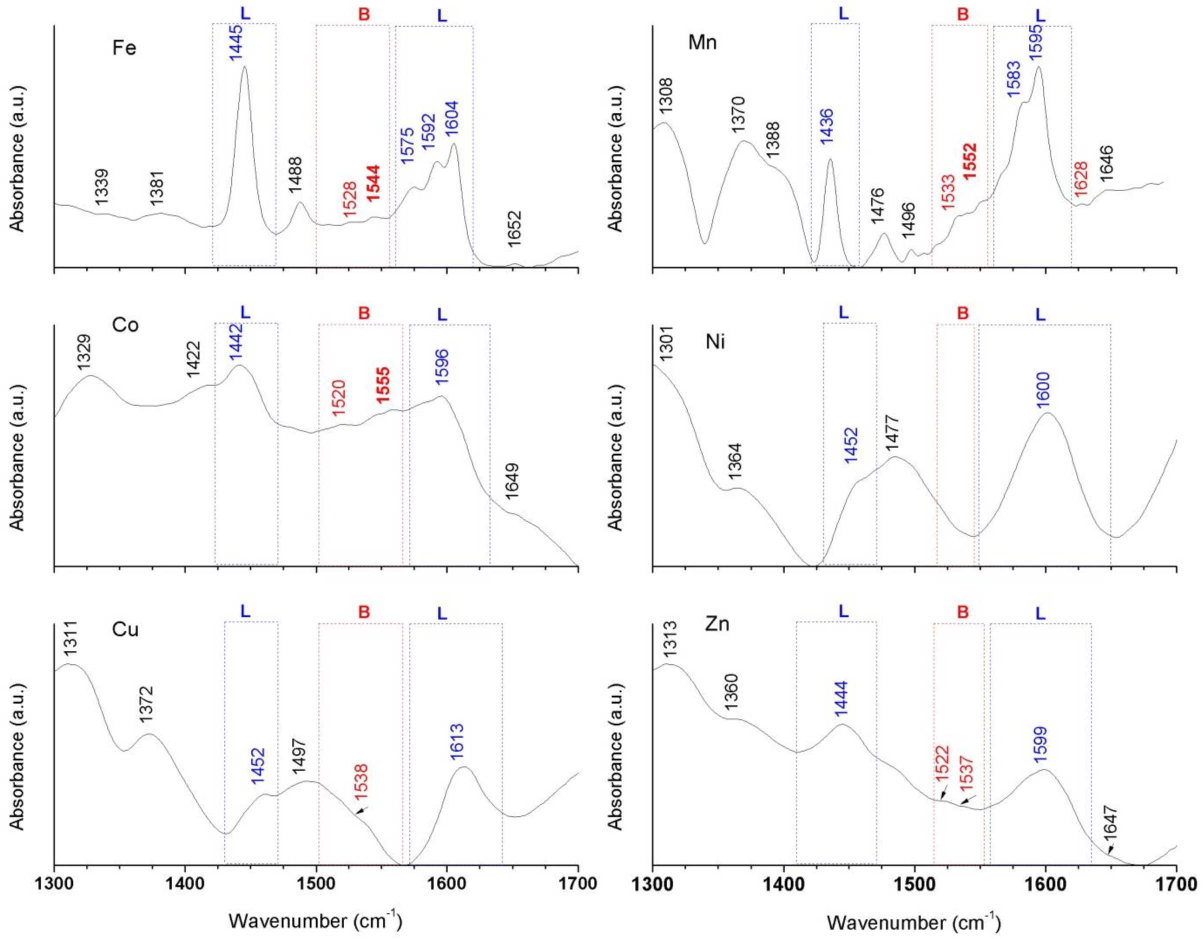

Fig S1 DRIFT spectra of pyridine adsorbed on the investigated solid samples

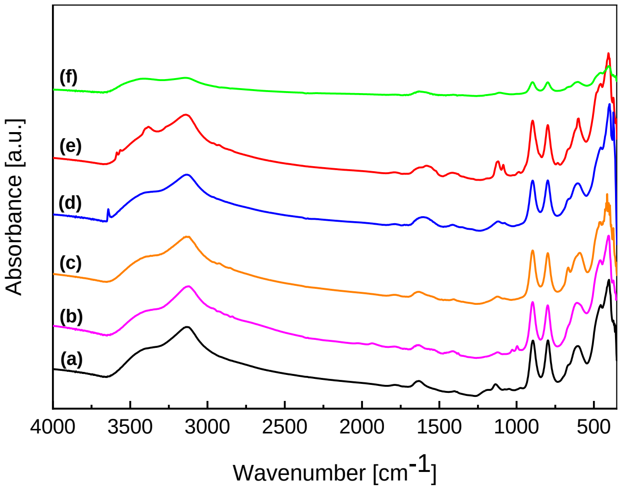

Fig S2 Infrared spectra of goethite (a), **Mn-composite** (b), **Co-composite** (c), **Ni-composite** (d), **Cu-composite** (e) and **Zn-composite** (f).

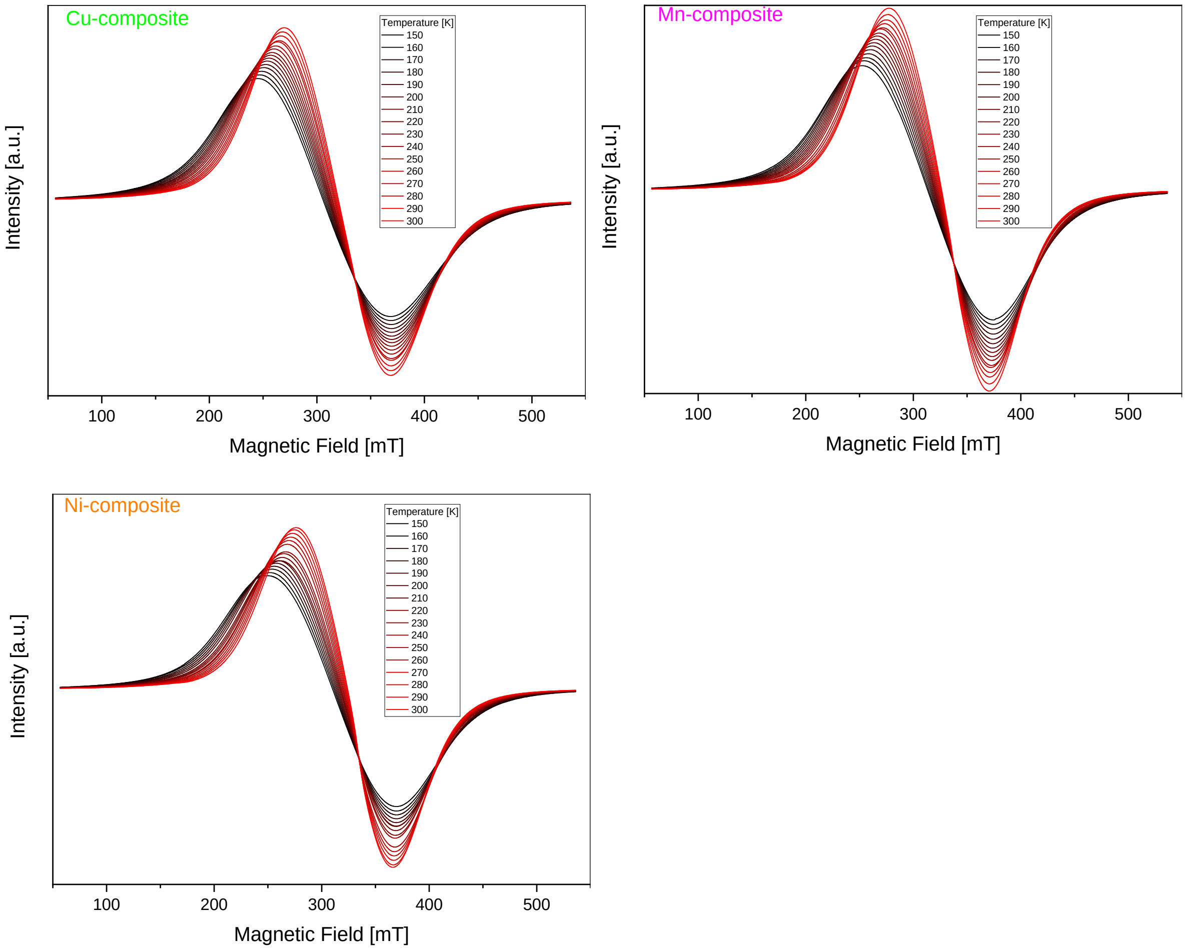

Fig S3 Temperature dependency of the EPR spectra for **Cu**, **Mn** and **Ni-composites**.
